# Pilates training improves 5-km run performance by changing metabolic cost and muscle activity in trained runners

**DOI:** 10.1371/journal.pone.0194057

**Published:** 2018-03-21

**Authors:** Paula Finatto, Edson Soares Da Silva, Alexandre B. Okamura, Bruna P. Almada, Henrique B. Oliveira, Leonardo A. Peyré-Tartaruga

**Affiliations:** Exercise Research Laboratory, Escola de Educação Física, Fisioterapia e Dança, Universidade Federal do Rio Grande do Sul, Porto Alegre, Rio Grande do Sul, Brazil; Fondazione Santa Lucia Istituto di Ricovero e Cura a Carattere Scientifico, ITALY

## Abstract

**Purpose:**

Strength training improves distance running economy and performance. This finding is based predominantly on maximal and explosive strength programmes applied to locomotor muscles, particularly on the lower limbs. It is not certain whether a minimization of metabolic cost (C_met_) and an improvement in running performance is feasible with strength training of the postural and trunk muscles.

**Methods:**

Using kinematic, neuromuscular and metabolic measurements of running at two different speeds before and after a 12-week Pilates training programme, we tested the hypothesis that core training might improve the running C_met_ and performance of trained runners. Thirty-two individuals were randomly assigned to the control group (CG, *n* = 16) or the Pilates group (PG, *n* = 16).

**Results:**

Confirming our hypothesis, a significant improvement (p<0.05) was observed for running performance in the PG (pre: 25.65±0.4 min; post: 23.23±0.4 min) compared to the CG (pre: 25.33±0.58 min; post: 24.61±0.52 min). Similarly, the PG (4.33±0.07 J.kg^-1^.m^-1^) had better responses than the CG (4.71±0.11 J.kg^-1^.m^-1^) during post-training for C_met_. These findings were accompanied by decreased electromyographic activity of the postural muscles at submaximal running intensities in the PG.

**Conclusions:**

Overall, these results provide a rationale for selecting strength training strategies that target adaptations on specific postural and locomotor muscles for trained distance runners.

## Introduction

From the cardiorespiratory perspective, running performance, particularly at long distances, depends on the interaction of different factors [1], including high maximum oxygen consumption (VO_2max_), the ability to sustain a high fraction of VO_2max_ for long periods, and the ability to move economically [2]. The latter parameter is designated as metabolic cost (C_met_) and corresponds to the oxygen consumption spent to move a certain distance by running at a submaximal intensity. Considering a group of runners with a similar body mass, an individual with a low C_met_ would spend less energy and consequently would have lower oxygen consumption (VO_2_) than a runner with a high C_met_ at a certain running speed [3,4].

A lower C_met_ may be achieved via aerobic endurance training programmes, aerobic endurance combined with strength training, and plyometric training [5,6]. Another aspect that may be related to C_met_ is muscle activation, particularly that of muscles of the trunk and lower limbs. Behm et al.[7] observed that a greater activation of the obliquus externus abdominis muscle and erector muscles of the upper and sacral spine is required during running for the control of movements and that the activation pattern of these muscles may be associated with better performance. For this reason, a specific training programme can promote greater stability, which would decrease necessary muscle recruitment and consequently positively affect C_met_ [8,9].

Pilates training (PT) has been widely used to strengthen trunk muscles. PT is based on six key principles: concentration, control, precision, flow, breathing, and centre of force [10]. The centre of force was originally designated powerhouse and refers to the extensor muscles of the spine and hip, the flexor muscles of the spine and hip, and the muscles of the pelvic floor [10]. The centre of force is strengthened to promote further stabilization of the hip and trunk and favour the integrity of the spine [11].

To the best of our knowledge, no previous studies have specifically addressed the effects of PT on running. However, training programmes for the stability of the core muscles, which correspond to the flexor and extensor muscles of the trunk, along with the deeper muscles that stabilize the trunk, have shown conflicting results when performed for six weeks. Stanton et al. [12] found significant improvements in core stability in team sport athletes after core training using Swiss balls; however, they found no significant differences in the activation of abdominal and extensor muscles of the spine, VO_2max_ or C_met_. By contrast, Sato and Mokha [9] found no significant improvement in the dynamic stability of trained runners after a core-training programme but found a significant decrease in the time of completion for a 5-km run.

Core training and PT aim to strengthen the muscles of the trunk and lower limbs. However, the principles inherent to PT are not used in core training, and these principles distinguish these two training modalities and can influence the results of PT. Specific training programmes can result in a better pattern of activation of the trunk muscles, which would provide more stable joints and reduce the need for co-contractions for stabilization. Consequently, these programmes could lead to decreased C_met_ and, in turn, improved running performance. We hypothesized that metabolic cost and trunk muscle activation will be reduced and, consequently, running performance may be improved. Therefore, it is essential to study the effects of strengthening the muscles of the centre of force by PT on C_met_ and on the muscle activation patterns and biomechanical parameters that could be indicative of improved C_met_ because this strategy can consequently increase running performance.

## Materials and methods

### Experimental design

To investigate the effects of mat PT in recreational runners, cardiorespiratory and neuromuscular adaptations were compared between a group that underwent running training combined with PT and a control group that underwent only running training. Both groups (Pilates and control) were trained for 12 weeks and were evaluated before and after the training period. The post-training evaluations were performed 72 hours after the last training session, and the subjects completed the evaluations within 10 days with at least 48 hours between the test sessions. The same assessors who were blinded to the training groups conducted the test sessions, and the same equipment was used in all of the sessions. The subjects were instructed to maintain their eating habits during the study period.

### Participants

Fifty-eight subjects were interviewed after placement of an advertisement about the study in a major newspaper in Porto Alegre, Brazil. The 32 enrolled volunteers were randomly assigned into two groups by electronic randomization: control group (CG; n = 16) (mean±SE, age: 18.44 ± 0.52 years; body mass: 73.64 ± 10.79 kg; height: 176.66 ± 9.89 cm, percent fat: 10.81 ± 2.49%) and Pilates group (PG; n = 16) (mean ± SE, age: 18.42 ± 0.51 years; body mass: 70.71±10.90 kg; height: 175.07 ± 8.06 cm, percent fat: 9.34±1.98%). During data collection, the CG lost three subjects. During the training period, one subject from the PG was excluded because his rate of absence from training was higher than 20%. Therefore, 15 subjects in the CG and 13 subjects in the PG completed all phases of the study. The inclusion criteria were as follows: male, practice of running for at least six months before the study, with experience in 5-km running races, age between 18 and 28 years, and absence of medical restrictions. The exclusion criteria were as follows: subjects with experience in Pilates and subjects with hormonal, metabolic, neuromuscular, and/or cardiac disorders. All the participants had a running experience of at most 9 months prior to the start of the study, with the main frequency of 2 times a week. Each individual signed a free and informed consent form. This study was conducted according to the Helsinki Declaration and was approved by the Ethics Committee of the Federal University of Rio Grande do Sul, Brazil under registration no. 965734.

### Procedures

#### Running training

Subjects from the CG and PG participated in a 12-week racetrack training programme (see the training program in the supplementary material, S1 Table). Two sessions per week were performed. The periodization of running training was based on the second ventilatory threshold (VT2) obtained in a maximal effort test on a treadmill with maximum oxygen consumption (VO_2max_) in a first session of data collection. Accordingly, the training periodization was based on the heart rate at VT2 (HR_VT2_) according to the intensity zones proposed by Daniels [13]: easy (E), 71–86%; moderate (M), 82–98%; threshold (T), 96–100%; and interval (I), 107–109% of HR_VT2_ (S1 Table). The training sessions were held at the three racetracks of the School of Physical Education of the Federal University of Rio Grande do Sul (Escola de Educação Física, Fisioterapia e Dança da Universidade Federal do Rio Grande do Sul).

#### Classical mat Pilates training

The classic mat PT programme lasted 12 weeks. The subjects from the PG underwent the running training described above in addition to two one-hour weekly sessions of PT performed on days alternate to the days of the running training. The organization of the session and the intensity and volume of training were in accordance with the Manual of the Pilates Method Alliance (California, USA). The sessions were organized into an initial section (execution of PT fundamentals), a main section (execution of PT exercises), and a final section (relaxation). During the initial section, the fundamentals of PT were performed, and the exercises were selected according to the training period. Classic mat PT consisted of three series, and the number of repetitions and sequences were defined as shown in Table 1.

**Table 1:** 12-week periodization of Pilates training.

	**Week 1**	**Weeks 2 to 6**	**Weeks 6 to 12**
Initial section	Fundamentals 1 to 7	Fundamentals 5 to 12	Fundamentals 13 to 17
Main section	Pre-Pilates	Basic Mat Pilates	Intermediate Mat Pilates
Final section	Relaxation	Relaxation	Relaxation
**Exercises that composed the various levels**			
**Fundamentals**	**Pre-Pilates**	**Basic Mat Pilates**	**Intermediate Mat Pilates**
1. Breathing	1. The Hundred	1.The Hundred	1. The Hundred
2. Imprinting	2. Roll Down	2. The Roll Up	2. The Roll Up
3. Pelvic Bowl	3. Roll Up	3. Single Leg Circles	3. Leg Circles
4. Knee Sway	4. Single Leg Circles	4. Rolling Like a Ball	4. Rolling Like a Ball
5. Knee Folds/Stirs	5. Rolling Like a Ball	5. Single Leg Stretch	5. Single Leg Stretch
6. Leg Slides	6. Single Leg Stretch	6. Double Leg Stretch	6. Double Leg Stretch
7. Spinal Bridging	7. Double Leg Stretch	7. Legs Up and Down	7. Single Straight Leg
8. Prone Hip Extension	8. Spine Stretch Forward	8. Spine Stretch Forward	8. Double Straight Leg
9. Cervical Nod		9. Saw	9. Criss-Cross
10. Nose Circles		10. Single Leg Kicks	10. Spine Stretch Forward
11. Head Float		11. Beats	11. Open Leg Rocker
12. Ribcage/Angel Arms		12. Double Leg Kicks	12. Corkscrew
13. Rotating Arms			13. Saw
14. Torso Twist			14. Neck Pull
15. Flight			15. Single Leg Kicks
16. Cat			16. Double Leg Kicks
17. Bowing			17. Neck Pull
			18. Side Kicks Series
			19. Teaser
			20. Seal

### Maximum amplitude of the electromyographic signal during MVIC

In a second session, for the evaluation of the maximum isometric amplitude of the electromyographic (EMG) signal of the aforementioned muscles, the procedures began with electrode placement and skin preparation on the muscle surfaces of interest [14]. Surface electrodes with a 15-mm total diameter (Meditrace^TM^, Mainsfield, Canada) were used in a bipolar configuration, whose inter-electrode distance of 2 cm [15].

After this procedure, the locations of electrode placement for the longissimus (LO), gluteus medius (GM), vastus lateralis (VL), biceps femoralis (BF) and latissimus dorsi (LD) were determined according to the recommendations of the SENIAM project (Surface ElectroMyoGraphy for the Non-Invasive Assessment of Muscles; [16]). In the obliquus internus abdominis (OI) muscle, the electrodes were placed two cm medially and inferiorly to the anterior superior iliac spine [17]. In the obliquus externus abdominis (OE) muscle, the electrodes were placed at mid-distance between the lower part of the rib cage and the anterior superior iliac spine [17]. The reference electrode was placed at the tuberosity of the tibia of the right leg. A level of resistance between the electrodes of up to 3000 Ω was accepted. After placement of the electrodes, the subjects were instructed to perform a warm-up by walking for 5 min on a treadmill. All of the subjects were instructed and encouraged to exert the maximum force in each isometric test against the mechanical strength of the Velcro strips to produce this force as fast as possible. Three measurements of the maximal voluntary isometric contraction (MVIC) were obtained in each muscle, with a duration of 5 s and an interval of 3 min between each measurement pre- and post-training and used to normalize the EMG activation during running in each evaluation period. To obtain muscle activation, the EMG signal was captured by two electromyographs (Miotool 400, Miotec, Porto Alegre, Brazil), with a sampling frequency of 2000 Hz in each channel, using Miograph software (Miotec, Porto Alegre, Brazil) for later analysis in SAD32 software (UFRGS, Porto Alegre, Brazil). The signal was filtered using a fifth-order Butterworth band-pass filter with cut-off frequencies between 20 and 500 Hz. After filtering, the plateau period of isometric activation for 1-s intervals was identified. The root mean square (RMS) value was obtained via Hamming windowing in 1-s intervals. The measurement with the highest RMS value was considered valid.

### EMG activation during running

Fifteen minutes after the completion of the MVIC measurements, the running protocol was initiated with two runs at speeds of 10 and 12 km.h^-1^ performed in random order for 7 minutes each. The EMG signal was recorded in the last minute of the protocol on a treadmill at each speed evaluated using the data acquisition software Miograph (Miotec, Porto Alegre, Brazil). The kinematic data used for evaluation of the EMG signal at the different stride phases were also obtained in the last minute of each speed evaluated by recording the run with a Casio (EXLIM-ZR1000) video camera at a sampling rate of 120 Hz. These data were aligned with the EMG data using a light signal that generates a spike in the EMG signal in the channel specified for the alignment. Subsequently, the files with data on running and MVICs were exported for analysis in SAD32 software (UFRGS, Porto Alegre, Brazil). For the analysis of EMG activation during the runs, the same signal filtering procedure as that used for MVIC was applied. The RMS curve was obtained via Hamming windowing in 0.1-second intervals. Subsequently, the RMS signal was shifted and clipped from the signal emitted by the alignment system, in agreement with the video analysis. The times corresponding to the three stride phases (pre-activation: 100 ms before the contact of the heel with the floor [18]; b) contact phase (contact of the heel with the ground until detachment of the heel from the ground); and c) swing phase (detachment of the heel from the ground until contact of the heel to the ground)) were then identified in the videos for clipping of the EMG signal obtained from five main strides. Subsequently, the mean time was calculated from these clippings to obtain the mean RMS value for each subject and for each stride phase. RMS values representative of EMG activation in each of the three phases were expressed as a percentage of MVIC. The pattern of EMG activation of the seven muscles analysed during the runs pre-and post-training in the CG and PG was analysed via a temporal analysis of the EMG signal in relation to time, considering that the normalized x-axis varied between 0 and 100% of the stride. For this purpose, the raw EMG signal was shifted according to the alignment system and was rectified and filtered using a fifth-order Butterworth low band-pass filter with a cut-off frequency of 10 Hz [19]. After filtering, the EMG signals from five main strides were obtained to calculate the mean curve. The mean curve of each subject was resampled in 200 points and exported to Excel (Microsoft, Redmond, USA) for the calculation of the mean curve between the subjects.

### Metabolic cost

The treadmill tests at both speeds were performed concurrently with the collection of EMG data. For this purpose, the subjects remained at rest for 15 min in the sitting position and at rest for 5 min in the orthostatic position to determine the at rest heart hate and VO_2_ for confirmation of the initiation of the test. The respiratory exchange ratio should be below 0.85 to ensure that the individual started from the same resting state in each phase of the test. Subsequently, a 5-min warm-up was performed on a treadmill and was immediately followed by two additional 7-min stages, at running speeds of 10 and 12 km.h^-1^. These speeds were randomized, respecting a 5-min interval between the runs or until the heart hate returned to resting levels. The VO_2_ values were collected in the last 4 min of each run, and the last 3 min were included in the analysis. Data were collected using a gas analyser model VO2000 (Medgraphics, Ann Arbor, USA). The W_met_ was considered the difference between the VO_2_ measured during exercise and the VO_2_ at rest, in relation to time. Because the unit of measure used was watts (W), this difference was multiplied by the energy coefficient (20.9 J.mL^-1^) and divided by the time in seconds (60 s). The metabolic cost values relative to the speeds of 10 km.h^-1^ (C_met10_) and 12 km.h^-1^ (C_met12_) were calculated by dividing W_met_ by the speed in m.s^-1^.

### Time of completion of the 5-km run

In a third session, a 5-km run was performed by all of the subjects in a single test to determine the total time of completion of the test. The 5-km time was controlled using timers and was confirmed by filming. The race was always held at the same time and with similar temperature and relative humidity conditions. All data can be seen in the supplementary material (S2 Table).

### Statistics

The comparisons of run performance variables, metabolic variables, muscle activation, and sample characteristics between the groups and time factors were performed using the generalized estimating equations model. Bonferroni’s complementary test was used to identify significant differences. The significance level was set at α<0.05, and the statistical package used was SPSS version 18.0 (IBM, Armonk, USA).

The sample size computation was based on data (C_met_ and performance) from Sato & Mokha [9] and Stanton et al. [12]. The software used was GPOWER version 3.1 (Power as 1-beta error probability: 95%; Effect size: 0.90; Error assumed as alpha: 0.05). After calculation, 26 subjects were indicated for allocation equally for each group, 13 subjects in CG group and 13 in PG group. We decided insert more subjects in each group, due to a possible sample loss. Therefore, the present study was initiated with 32 individuals.

## Results

### Participant baseline characteristics

The sample characterization data are shown in Table 2. No significant differences in this section were observed between the groups in the pre-training period.

**Table 2:** Mean (standard deviation) age, height, body mass, body fat, lean mass, maximal oxygenuptake (VO_2max_), and speed at the second ventilatory threshold (VT2) in the pre-training period.

Variable	Group		
	Control group (n = 16)	Pilates group (n = 15)	p-value
**Age (years)**	18.44 (0.52)	18.42 (0.51)	0.996
**Height (cm)**	176.66 (9.89)	175.07 (8.06)	0.404
**Body mass (kg)**	73.64 (10.79)	70.71 (10.90)	0.391
**Body fat (%)**	10.81 (2.49)	9.34 (1.98)	0.205
**Lean mass (%)**	49.82 (2.26)	50.54 (2.40)	0.583
**Speed at VT2 (km.h**^**-1**^**)**	14.44 (1.33)	14.21 (1.05)	0.837
**VO**_**2max**_ **(mL.kg**^**-1**^**.min**^**-1**^**)**	51.26 (5.43)	51.75 (7.55)	0.926

### Running performance and respiratory variables

The variables running time, VO_2max_, C_met10_, and C_met12_ were not significantly different between the groups in the pre-training period. In the post-training period, the PG had significantly higher VO_2max_ values (p<0.001), a significantly shorter 5-km running time (p<0.001), and a significantly lower C_met12_ (p = 0.019).

For the time factor, significant differences were found in both groups for all of the variables evaluated (Table 3).

**Table 3:** Effect of running training and running training combined with Pilates on performance and respiratory variables. Data Represent the Mean Values (Standard Error) for 5-km Running Time, Maximum Oxygen Consumption (VO_2max_), Metabolic Cost at 10 km.h^-1^ (C_met10_), Metabolic Cost at 12 km.h^-1^ (C_met12_), Speed at the Second Ventilatory Threshold (VT2), and Oxygen Consumption at the Second Ventilatory Threshold (VO_2_ VT2).

Variable	Group	Period		Effect of time	Effect of group	Interaction group x time
		Pre-training	Post-training	p-value	p-value	p-value
**5-km running time**	CG	25.33 (0.58)	24.61 (0.52)*	<0.001	0.441	<0.001
(min)	PG	25.65 (0.44)	23.23 (0.40)*^a^			
**VO**_**2max**_	CG	51.32 (1.20)	53.72 (1.58)*	<0.001	0.204	<0.001
(mL.kg^-1^.min^-1^)	PG	51.8 (1.73)	58.53 (1.59)*^a^			
**C**_**met10**_	CG	4.27 (0.09)	3.85 (0.13)*	<0.001	0.868	0.923
(J.kg^-1^.m^-1^)	PG	4.26(0.09)	3.82 (0.08)*			
**C**_**met12**_	CG	5.22 (0.08)	4.71 (0.11)*	<0.001	0.014	0.019
(J.kg^-1^.m^-1^)	PG	5.00 (0.10)	4.33 (0.07)*^a^			

*Significant difference between pre- and post-training

^a^ significant difference between the groups in post-training

### Electromyographic variables

#### Maximal voluntary isometric contraction

The comparisons between the training periods indicated that the MVIC of the OE, OI, LO, BF, and GM muscles increased significantly in only the PG whereas the activation of the VL muscle increased significantly in both the CG and PG between pre- and post-training (Table 4). In addition, no significant differences in MVIC were found between the CG and PG in pre-training. In post-training, the MVIC of the OE, OI, LO, and GM muscles was significantly higher in the PG than in the CG.

**Table 4:** Effects of running training (CG) and running training combined with Pilates (PG) on maximal voluntary isometric contraction (MVIC) in millivolts (mV) of the obliquus externus abdominis (OE), obliquus internus abdominis (OI), vastus lateralis (VL), longissimus (LO), biceps femoris (BF), gluteus medius (GM), and latissimus dorsi (LD) muscles.

Variable	Group	Period		Effect of time	Effect of group	Interaction group x time
		Pre-training	Post-training	p-value	p-value	p-value
**OE MVIC**	CG	233.00 (28.41)	229.36 (41.75)	0.005	0.048	0.047
(mV)	PG	249.87 (22.12)	316.95 (26.70)*^a^			
**OI MVIC**	CG	527.14 (60.5)	510.52 (71.19)	0.03	0.037	0.006
(mV)	PG	550.69 (60.85)	685.48 (73.46)*^a^			
**VL MVIC**	CG	425.53 (26.82)	483.16 (36.73)*	0.032	0.193	0.138
(mV)	PG	432.39 (40.79)	561.93 (46.61)*			
**LO MVIC**	CG	284.31 (17.51)	285.79 (15.25)	0.012	0.027	0.016
(mV)	PG	299.39 (21.31)	371.22 (21.49)*^a^			
**BF MVIC**	CG	379.40 (29.5)	452.78 (37.11)*	<0.001	0.559	0.034
(mV)	PG	370.47 (30.56)	510.20 (27.59)*			
**GM MVIC**	CG	471.15 (39.47)	529.03 (53.10)	0.006	0.007	0.040
(mV)	PG	450.12 (33.60)	587.68 (45.61) *^a^			
**LD MVIC**	CG	348.14 (28.00)	376.24 (32.59)	0.502	0.660	0.745
(mV)	PG	376.05 (32.86)	385.81 (47.95)			

*Significant difference between pre- and post-training

^a^ significant difference between the groups in post-training

#### Muscle activity during running

The data on muscle activation during the stride phases, presented as a percentage of the MVIC, indicated a distinct behaviour in relation to the remaining variables analysed. In the pre-training period, significant differences in the level of activation of the OE and BF muscles were found in the swing phase at 10 km.h^-1^ (p = 0.018 and 0.048, respectively) and for the VL (p = 0.024) and BF (p = 0.26) muscles at 10 km.h^-1^ in the pre-activation phase.

#### Obliquus externus abdominis

**Fig 1:**
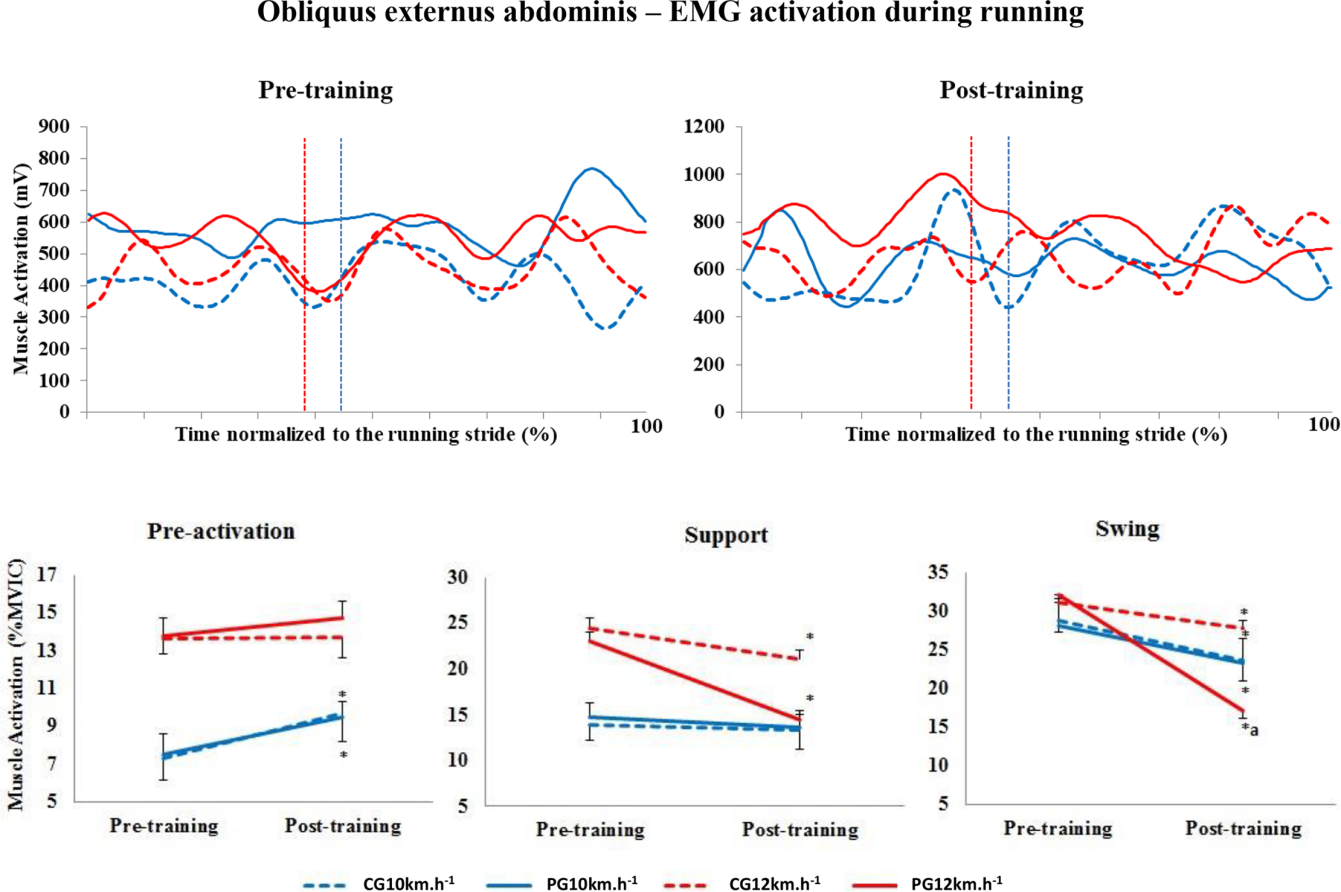
Upper panels, mean pattern of activation of the obliquus externus abdominis muscle (mV). Lower panels, mean (± standard error) muscle activation in the three stride phases, presented as a percentage of the MVIC. Red lines represent 12 km.h^-1^, and blue lines represent 10 km.h^-1^. Dotted lines represent the control group (CG), and solid lines represent the Pilates group (PG). The vertical dotted lines indicate the end of the contact phase. * Significant difference between pre- and post-training; ^a^ significant difference between groups (p<0.005).

A significant increase in the level of activation of the OE muscle was found in the pre-activation phase between the training periods at 10 km.h^-1^ (p = 0.022) (Fig 1). However, no differences in the level of activation of this muscle were found between groups (p = 0.983). In addition, at 10 km.h^-1^, the percentage of muscle activation between the training periods decreased only in the swing phase in both groups (p = 0.002).

The percentage of muscle activation significantly decreased in both groups in the support (p<0.001) and swing (p<0.001) phases at 12 km.h^-1^. Moreover, in the swing phase post-training, the level of activation was lower in the PG compared to the CG (p = 0.009).

#### Obliquus internus abdominis

**Fig 2:**
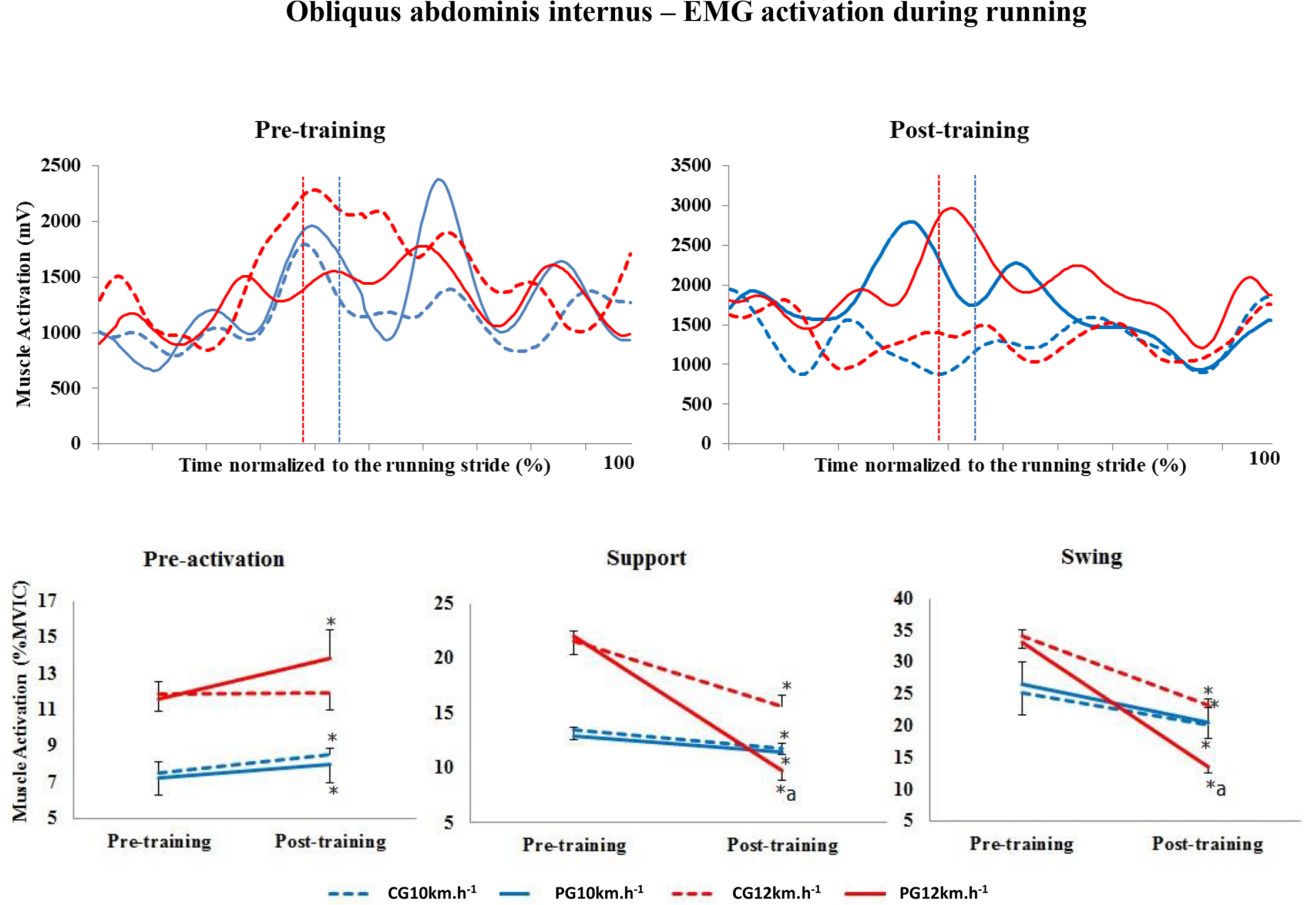
Upper panels, mean activation pattern of the obliquus internus abdominis muscle (mV). Lower panels, mean (± standard error) muscle activation in the three stride phases, presented as a percentage of the MVIC. Red lines represent 12 km.h^-1^, and blue lines represent 10 km.h^-1^. Dotted lines represent the control group (CG), and solid lines represent the Pilates group (PG). The vertical dotted lines indicate the end of the contact phase. * Significant difference between pre- and post-training; ^a^ significant difference between groups (p<0.005).

At 10 km.h^-1^, the level of activation of the OI muscle increased significantly in the pre-activation stage between pre- and post-training in both groups (p = 0.009) (Fig 2). At the speed of 12 km.h^-1^, the level of activation increased only in the PG in the pre-activation phase between pre- and post-training (p = 0.01); however, the level of muscle activation in the PG was lower than that in the CG (p = 0.003).

In the support and swing phases, the percentage of muscle activation decreased significantly between pre- and post-training at both speeds in both groups (p<0.001). At 12 km.h^-1^ in post-training, the level of activation in the PG was significantly lower than that in the CG (p = 0.01).

#### Vastus lateralis

**Fig 3:**
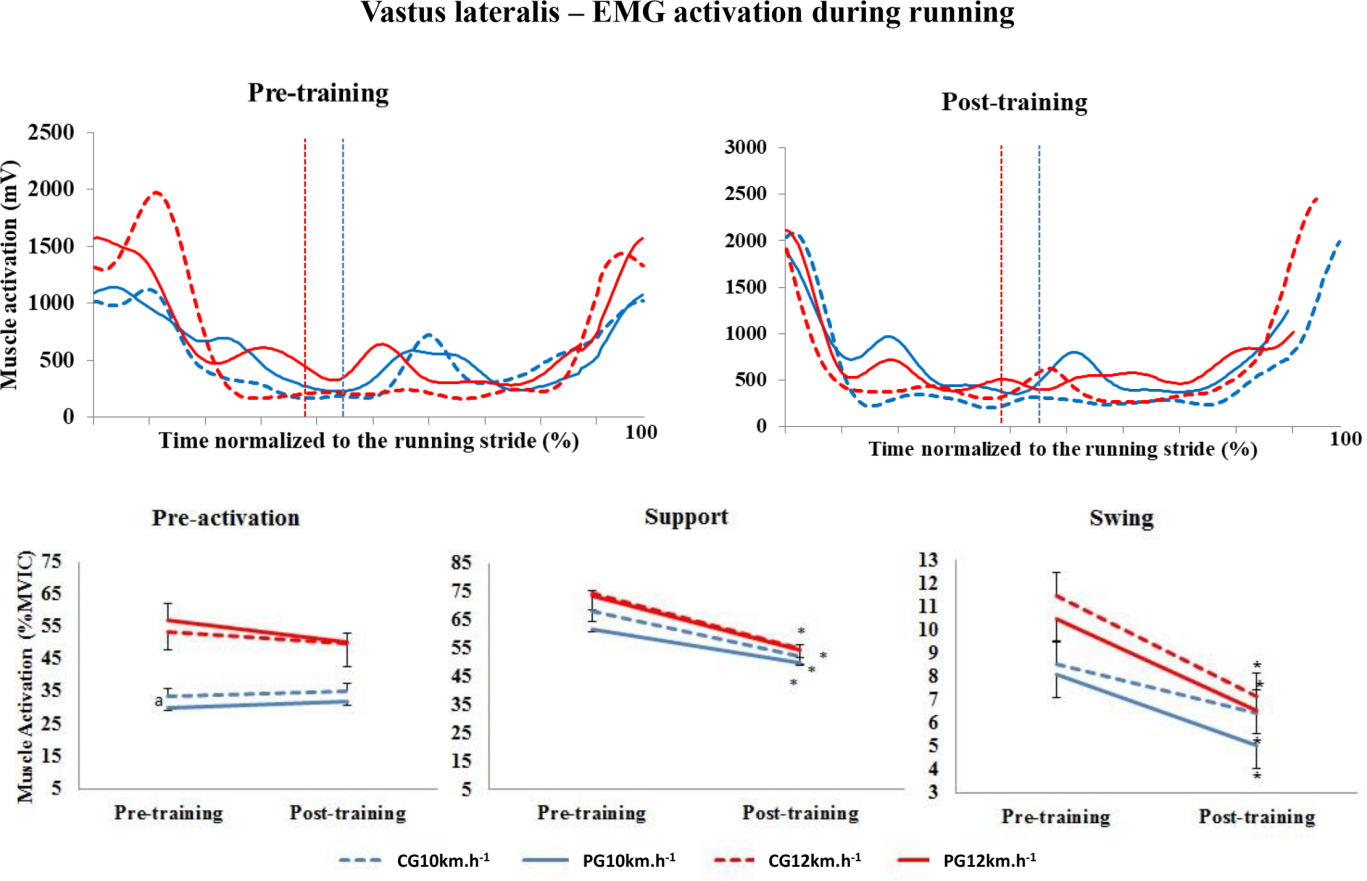
Upper panels, mean activation pattern of the vastus lateralis muscle (mV). Lower panels, mean (± standard error) muscle activation in the three stride phases, presented as a percentage of the MVIC. Red lines represent 12 km.h^-1^, and blue lines represent 10 km.h^-1^. Dotted lines represent the control group (CG), and solid lines represent the Pilates group (PG). The vertical dotted lines indicate the end of the contact phase. * Significant difference between pre- and post-training; ^a^ significant difference between groups (p<0.005).

The results for the VA muscle are shown in Fig 3. In the pre-activation phase (group factor, p = 0.273; time factor, p = 0.260) and swing phase (group factor, p = 0.551; time factor, p = 0.565), there were no significant differences in muscle activation in the conditions analysed. However, in the support phase, there was a significant decrease in the level of activation between the two training periods in all of the cases analysed (p<0.001). For the VA muscle in particular, there were no differences between the groups in any stride phases.

#### Longissimus

**Fig 4:**
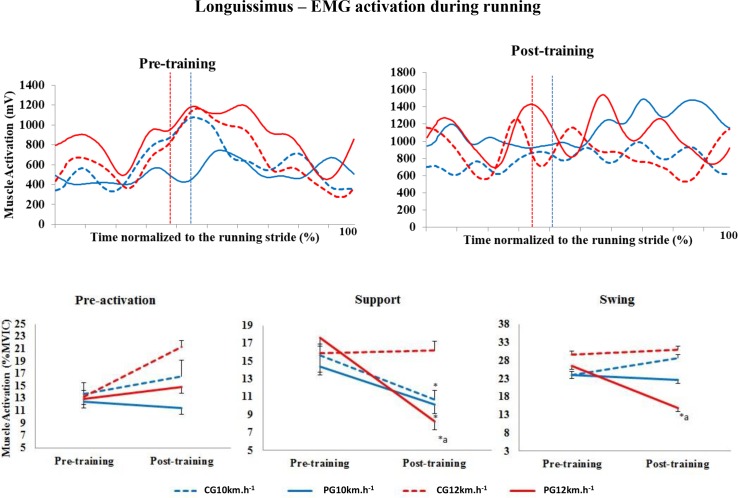
Upper panels, mean activation pattern of the longissimus muscle (mV). Lower panels, mean (± standard error) muscle activation in the three stride phases, presented as a percentage of the MVIC. Red lines represent 12 km.h^-1^, and blue lines represent 10 km.h^-1^. Dotted lines represent the control group (CG), and solid lines represent the Pilates group (PG). The vertical dotted lines indicate the end of the contact phase. * Significant difference between pre- and post-training; ^a^ significant difference between groups (p<0.005).

There were no significant differences in the level of activation of the LO muscle in the pre-activation phase in the conditions analysed. In the support phase at 10 km.h^-1^, the level of activation decreased in both groups (p = 0.001) (Fig 4). At 12 km.h^-1^, the level of activation decreased only in the PG between pre- and post-training (p = 0.003). Furthermore, in post-training, muscle activation in the PG was significantly lower than that in the CG (p = 0.002).

In the swing phase at 10 km.h^-1^, there were no significant differences in the level of activation of the LO between groups (p = 0.630) or between training periods (p = 0.364). At 12 km.h^-1^ in post-training, the level of activation of this muscle in the PG was significantly lower than in pre-training (p<0.001) and was significantly lower than in the CG (p = 0.005).

#### Biceps femoris

**Fig 5:**
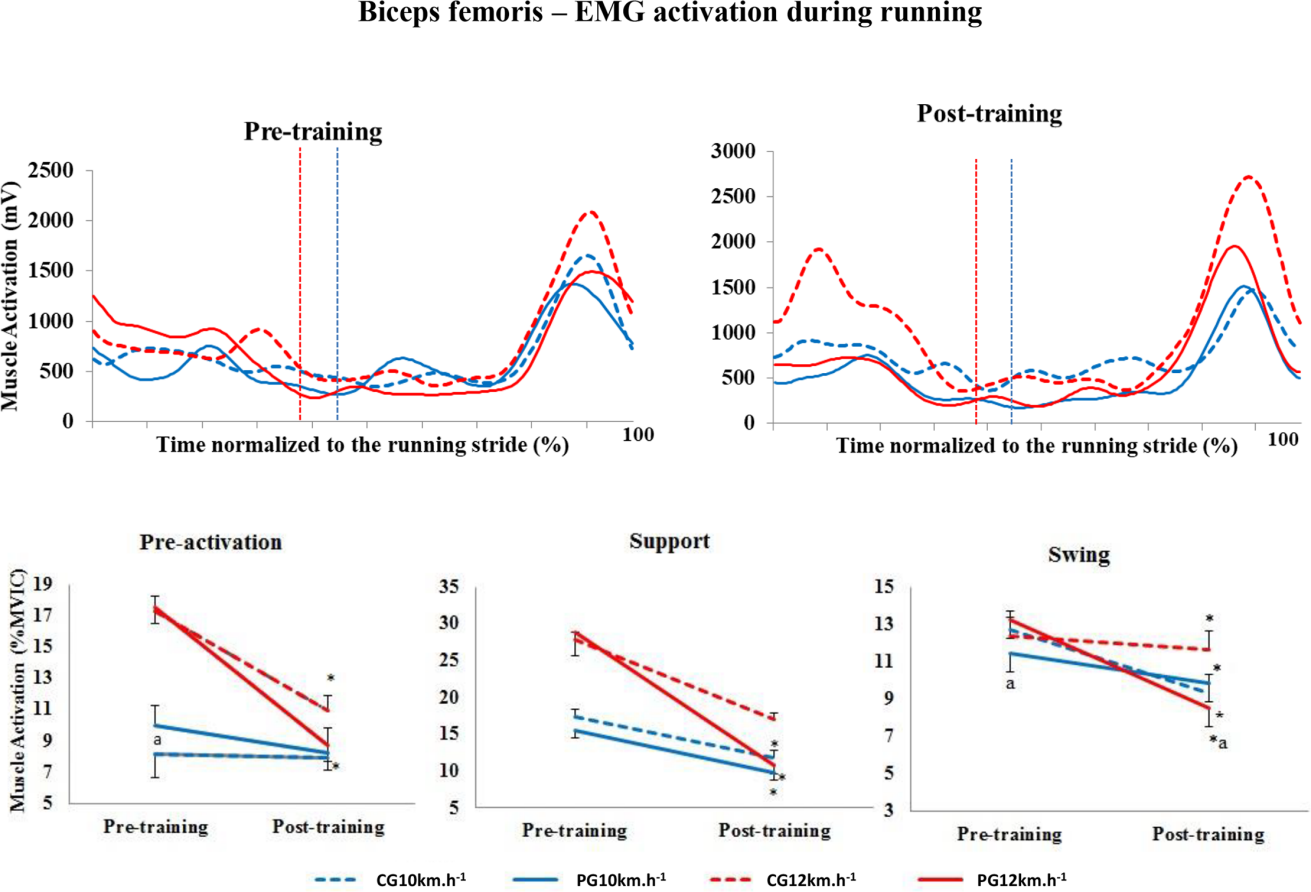
Upper panels, mean activation pattern of the biceps femoris muscle (mV). Lower panels, mean (± standard error) muscle activation in the three stride phases, presented as a percentage of the MVIC. Red lines represent 12 km.h^-1^, and blue lines represent 10 km.h^-1^. Dotted lines represent the control group (CG), and solid lines represent the Pilates group (PG). The vertical dotted lines indicate the end of the contact phase. * Significant difference between pre- and post-training; ^a^ significant difference between groups (p<0.005).

In the pre-activation phase at 10 km.h^-1^, there were no significant differences in the level of activation of the BF muscle between pre- and post-training (p = 0.498) (Fig 5). However, in the pre-activation phase at 12 km.h^-1^, the level of activation of this muscle decreased significantly in both groups (p<0.001).

In the support phase at 10 km.h^-1^ (p<0.001) and 12 km.h^-1^ (p<0.001), the level of activation of the BF decreased significantly between pre- and post-training regardless of the group evaluated. In the swing phase, the level of activation decreased significantly at the two speeds and in both groups.

#### Gluteus medius

**Fig 6:**
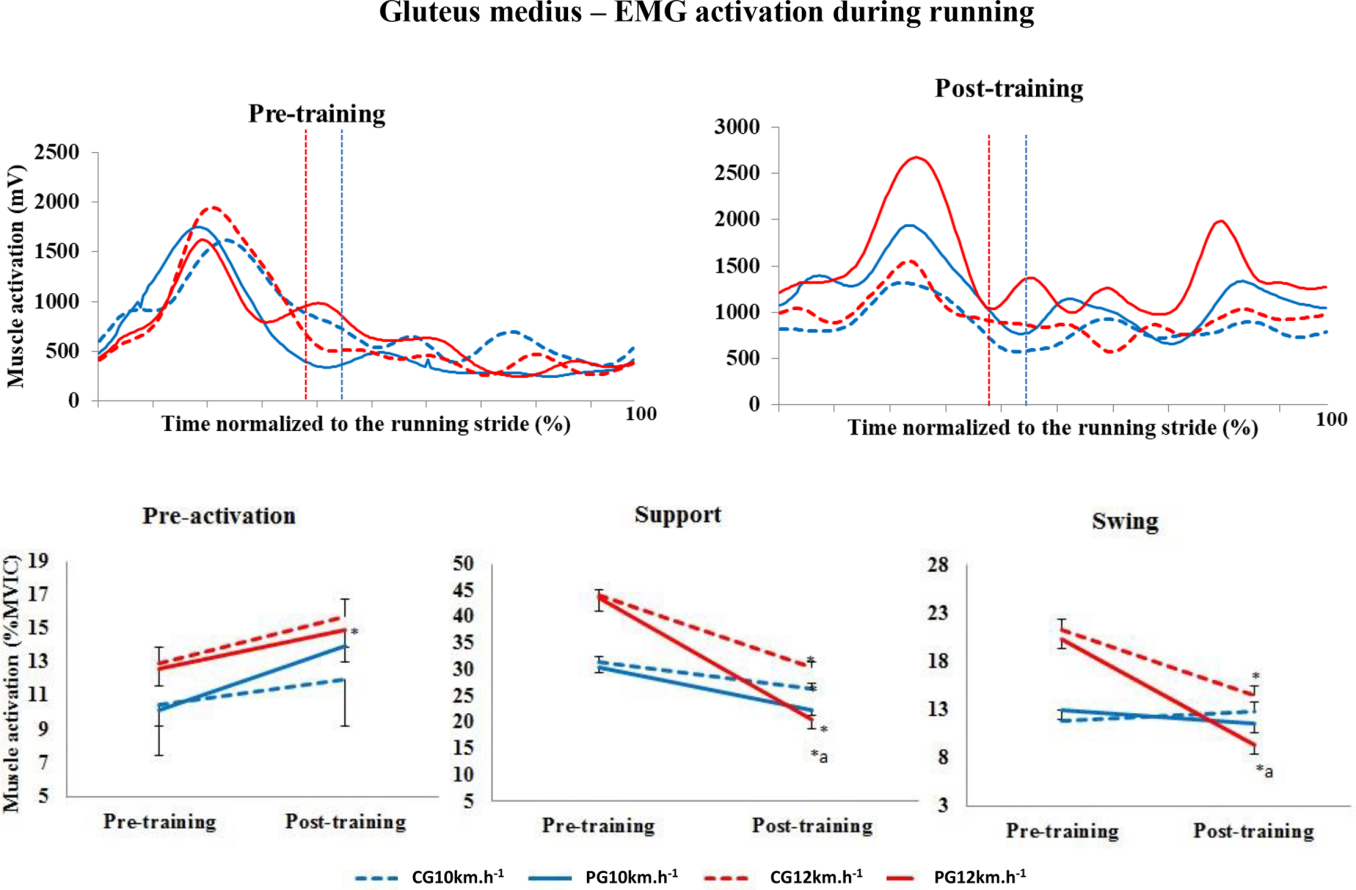
Upper panels, mean activation pattern of the gluteus medius muscle (mV). Lower panels, mean (± standard error) muscle activation in the three phases, presented as a percentage of the MVIC. Red lines represent 12 km.h^-1^, whereas blue lines represent 10 km.h^-1^. Dotted lines represent the control group (CG), and solid lines represent the Pilates group (PG). The vertical dotted lines indicate the end of the contact phase. * Significant difference between pre- and post-training; ^a^ significant difference between groups (p<0.005).

In the pre-activation phase at 10 km.h^-1^ and 12 km.h^-1^, no significant differences in the activation of the GM muscle were observed (group factor, p = 0.841, time factor, p = 0.083; group factor, p = 0.686, time factor, p = 0.081, respectively). In the support phase at 10 km.h^-1^ (p = 0.003) and 12 km.h^-1^ (p<0.001), the percentage of muscle activation decreased in both groups and at both speeds between the two training periods. At 12 km.h^-1^ in post-training, the percentage of muscle activation in the PG was significantly lower than that in the CG (p = 0.005). In the swing phase at 10 km.h^-1^, there were no significant differences in the percentage of muscle activation considering the time factor (p = 0.968) and group factor (p = 0.712). However, at 12 km.h^-1^, the level of activation of this muscle decreased in the CG and PG considering the time factor (p<0.001), and muscle activation in the PG was significantly lower than in the CG (p = 0.005) in post-training (Fig 6).

#### Latissimus dorsi

**Fig 7:**
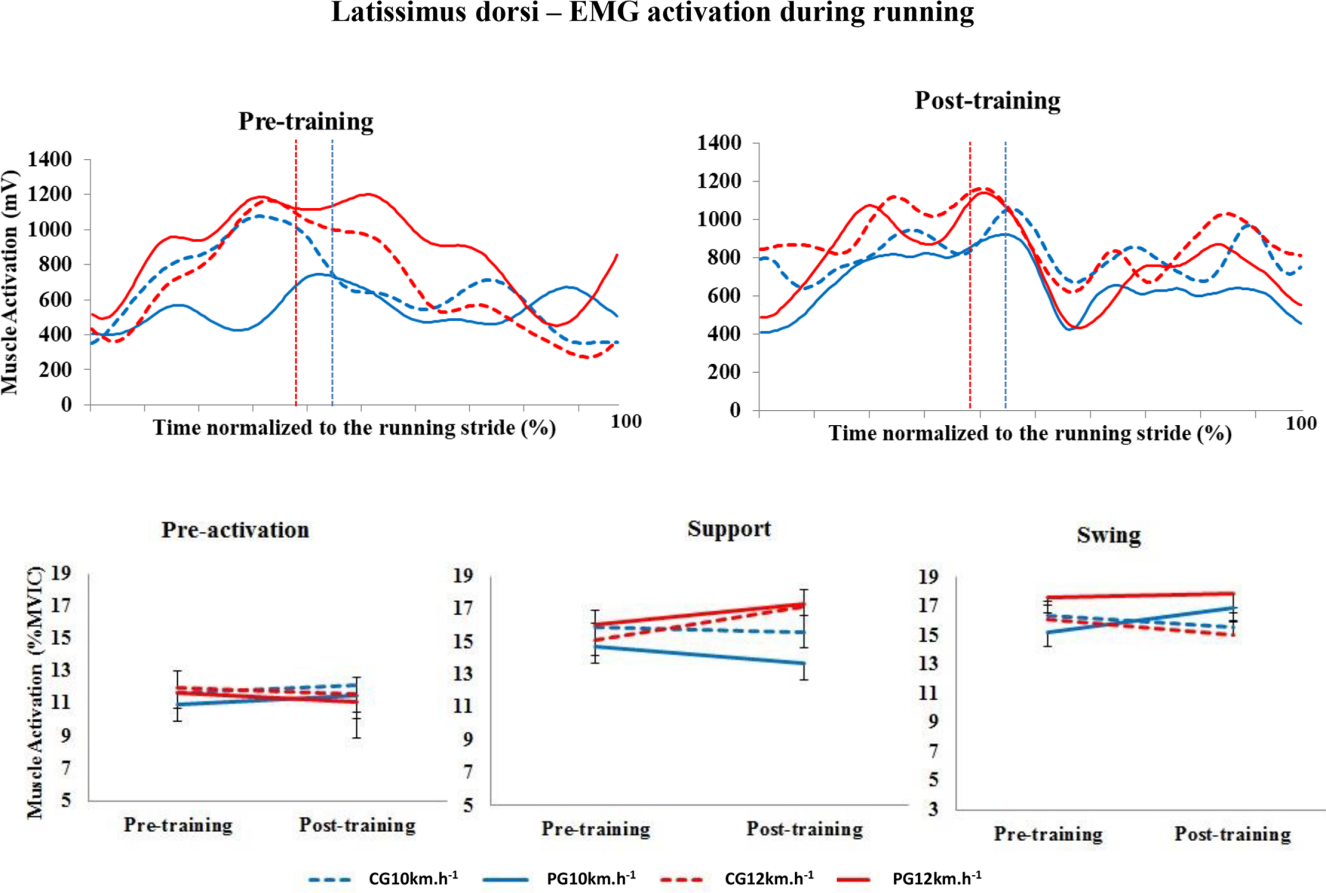
Upper panel, mean activation pattern of the latissimus dorsi muscle (mV). Lower panel, mean (± standard error) muscle activation in the three stride phases, presented as a percentage of the MVIC. Red lines represent 12 km.h^-1^, and blue lines represent 10 km.h^-1^. Dotted lines represent the control group (CG), and solid lines represent the Pilates group (PG). The vertical dotted lines indicate the end of the contact phase. * Significant difference between pre- and post-training; ^a^ significant difference between groups (p<0.005).

No significant differences were observed in the level of activation of the LD muscle in any of the running stride phases evaluated (Fig 7).

## Discussion

The results support our hypotheses that distance running performance is enhanced after a 12-weeks Pilates training programme. The improvements in performance are accompanied by a critical reduction on C_met_ and trunk muscle activation. This suggests that distance runners are able to transfer effective gains from a slow-type core strength training method to the running movement.

There is great interest in the mechanisms capable of minimizing energy expenditure during running because these mechanisms play an essential role in the search for strategies to improve performance. From the mechanical point of view, the "mass-spring” model reflects the occurrence of storage and release of elastic energy during running, which helps minimize the expenditure of metabolic energy [20]. Therefore, changes in this mechanism could affect performance and improve running economy (RE) [4].

Hoff et al.[21] concluded that shorter contact time with the ground is accompanied by a longer time at a lower level of muscle activation, indicating lower metabolic demand at the same submaximal speed.

Therefore, lower metabolic demand in the muscles is dependent on a number of factors, including the activation level of the task. According to a recent model by Miller et al.[22] on energy minimization during running, the decrease in muscle activity is the primary strategy to generate greater energy economy during running. This model was built using the speed of 3.76 m.s^-1^, which is similar to the highest speed evaluated in this study (12 km.h^-1^) and supports the results found herein.

In addition to the decrease in C_met_ at both speeds in both study groups as a result of the training applied, we found an overall decrease in the percentage of muscle activation at the same speed in post-training. Moreover, the PG displayed a significantly greater decrease in C_met12_ and in the 5-km run performance compared to the CG. This decrease was accompanied by a higher VO_2max_ and a further decrease in the level of activation of the OE (Δ6.77% in the swing phase,), OI (Δ5.84% in the support phase and Δ9.73% in the swing phase), LO (Δ8.00% in the support phase and Δ16.03% in the swing phase), and GM (Δ9.81% in the support phase and Δ5.05% in the swing phase) muscles compared to the CG at 12 km.h^-1^, in accordance with the model of Miller et al.[22].

**Fig 8:**
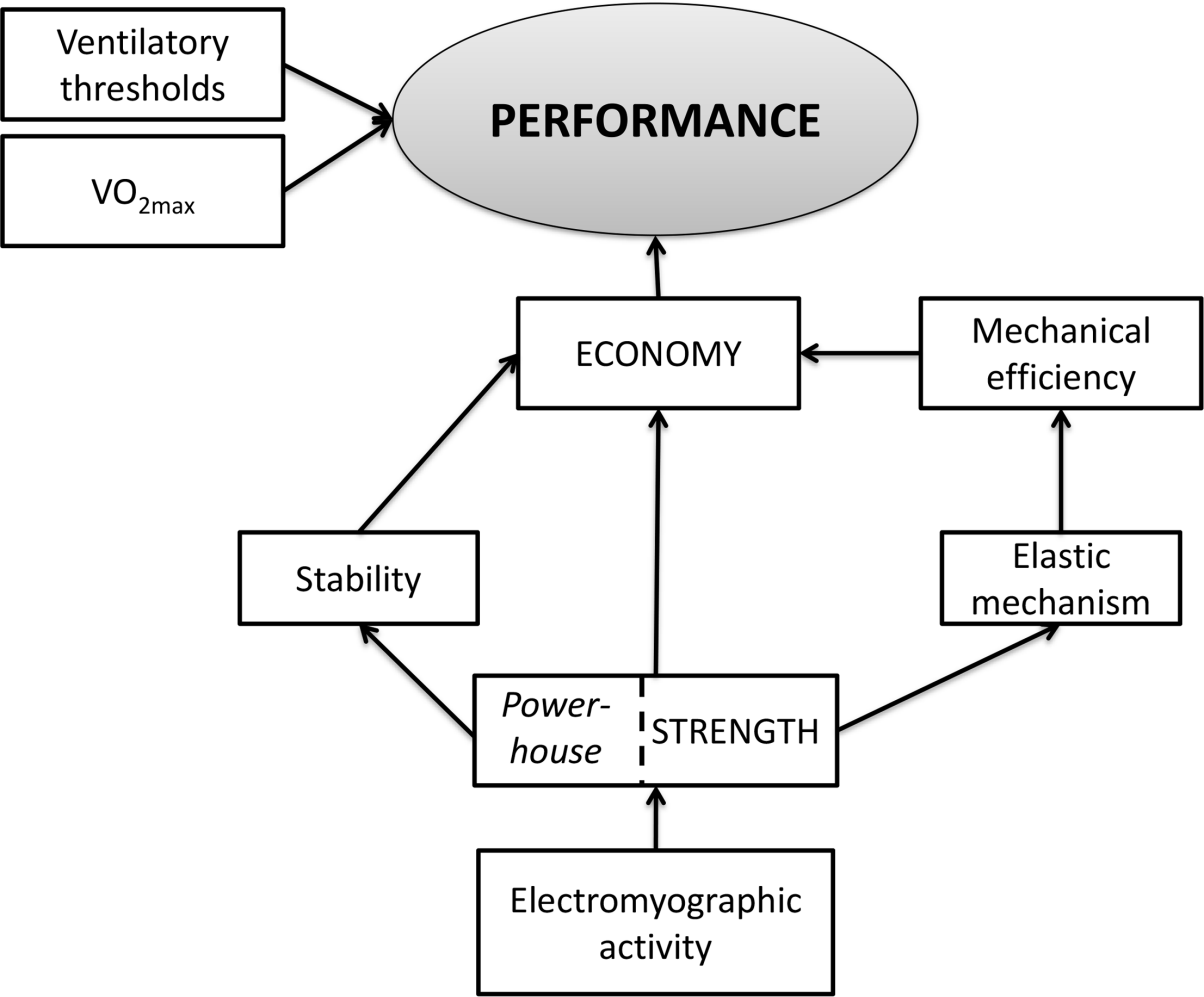
Schematic drawing of the performance model proposed in this study [2,3,20,22].

Therefore, if better performance in the 5-km run can be determined by a higher VO_2max_, the ability to sustain a higher fraction of VO_2max_, and a better RE, and because the decrease in the percentage of muscle activation optimizes energy minimization during running [21,22], our findings suggest the presence of a correlation between a 12-week training programme of classic PT and the mechanisms capable of minimizing energy during running, thus contributing to improved performance (Fig 8).

When analysed in isolation, the decreased percentage of muscle activation found during running in both groups at 10 and 12 km.h^-1^ can be explained by the so-called "neuromuscular economy" [23], which is defined as the decrease in the number of motor units recruited when considering a situation involving a similar submaximal task. This mechanism explains in part the results of the present study, in which the same running speeds were analysed in pre- and post-training.

Besides, the running training performed by the CG decreased the percentage of muscle activation at both speeds between pre- and post-training, and the decrease in the PG was significantly stronger. These results indicate the possible presence of neuromuscular economy, particularly when analysed together with the results for the MVIC. The maximum amplitude of the EMG signal in the VAS muscle was significantly higher post-training in both groups. However, the level of activation of the OA, LO, OI, and GM muscles increased significantly only in the PG, who underwent special training for these muscles.

The increase in the maximum amplitude of the MVIC along with the higher VO_2max_ found in post-training may decrease the relative loads, which correspond to the speeds in pre-training and justify the decreased recruitment of motor units during the performance of the same task in the post-training period. This hypothesis would explain the findings in the PG, who showed a stronger decrease in the percentage of muscle activation during running and shorter 5-km run completion time compared to the CG.

The reduction in the time of completion of this run, the improvement in C_met12_, and the greater decrease in the percentage of EMG activation in the PG during running appear to be associated with the control and stabilization of the lumbopelvic region. The correlation between EMG activity and stability is well established in the literature. In fact, findings empirically show that the neural system responds to changes in spinal stability [24] and gives support to the adaptive process model on motor learning [25]. The control of the trunk is an important factor for C_met_, and leg movements are closely associated with lumbopelvic movements; thus, the latter depend on the stiffness of the abdominal muscles [3,25].

Therefore, the increase in running speed would cause more lumbopelvic movements and consequently greater instability, which would require greater neuromuscular control to achieve stability during cyclical movements such as running [8,26]. In turn, this increased neuromuscular demand for stabilization of the lumbopelvic region appears to be associated with a greater contribution of concentric activations—which are more energy-consuming than eccentric and isometric activations—and reinforces the fact that an unstable system is also less economical [25,27].

From this perspective, lumbopelvic stabilization is one of the aims of Pilates. Among its guiding principles, breathing [28] and centring [29] have been shown to stimulate the deep abdominal muscles responsible for stabilization, including the rectus abdominis muscles, OI, OE, and transversus abdominis [8]. In this respect, Phrompaet et al.[30] evaluated the effects of PT in the control of lumbopelvic movements. At the end of eight weeks of training, the authors found that 65% of the subjects in the Pilates group passed the lumbopelvic stability test after four weeks of training, and 85% passed the test after eight weeks of training, whereas none of the subjects in the control group passed the test. The authors indicate that the improved recruitment of abdominal muscles during PT appears to help develop the strength of these muscles, leading to improved stability. However, EMG activity was not evaluated in that study.

Sato and Mokha [9] evaluated a six-week core-training programme and found improvement in a 5-km run completion time. The run completion time decreased significantly in the experimental group (from 29.29±2.38 to 28.42±2.23 min) but did not decrease in the control group. In addition, Stanton et al. [12] evaluated participants after six weeks of core training with a Swiss ball and found a significant improvement in lumbopelvic stability; however, no differences were found in running economy, VO_2max_, or in the EMG activity of the trunk muscles.

By contrast, in the present study, the run completion time decreased from 25.33±0.58 to 24.61±0.52 min in the CG and from 25.65±0.44 to 23.23±0.40 min in the PG. In addition, the PG had an improvement in VO_2max_ (from 51.8±1.73 mL.kg^-1^.min^-1^ in pre-training to 58.53±1.59 mL.kg^-1^.min^-1^ in post-training, p<0.001) and C_met12_ (from 5.0±0.10 J.kg^-1^.m^-1^ in pre-training to 4.33±0.07 J.kg^-1^.m^-1^ in post-training, p<0.001) and a decrease in the percentage of EMG activation of the trunk muscles.

However, despite the conflicting results with the literature with regard to core training, the present study is distinguished by the duration of the training period. In the cited studies, only a six-week training programme was conducted whereas the present study utilized a 12-week training programme in both groups, and this longer training may have contributed to the results obtained. Moreover, unlike core training, PT should be performed considering its principles, which can help increase muscle activation to higher levels [28,29].

In conclusion, PT significantly improved the 5-km run performance. This improved performance is associated with the optimization of mechanisms capable of minimizing energy expenditure. That is, a lower percentage of EMG activation of the trunk muscles during running as a result of strength gain. Therefore, the greater running economy seems to be positively influenced the 5-km run performance in recreational runners.

## Conclusions

The results of this study indicate that PT can be incorporated into the training programmes of recreational runners to improve running performance and VO_2max_ and to strengthen trunk muscles. In addition, in situations in which the development of aerobic power is limited by cardiac or pulmonary capacity, PT may improve performance at a lower metabolic cost by decreasing muscle demand during unnecessary pelvic movements and improve other health-related aspects, including a lower risk of injury. However, little is known about the effects of PT on mechanisms that minimize energy expenditure, mechanical parameters, and their correlation with running performance. Therefore, further studies are necessary to elucidate these relationships.

## Supporting information

S1 Table 12-week periodization of running training using the following intensity scores: E, Easy; M, Moderate; T, Threshold; and I, Interval, as a function of the heart rate at VT2. (DOCX)

S2 Table General dataset. (XLSX)

## Abbreviations

Anova: Analysis of variance
BF: biceps femoralis
C_met_: metabolic cos
C_met10_: metabolic cost at 10 km.h^-1^
C_met12_: metabolic cost at 12 km.h^-1^
CG: control group
E: Easy
EMG: Electromyographic
GM: gluteus medius
HR_VT2_: heart rate at second ventilatory threshold
LD: latissimus dors
LO: Longissimus
I: Interval
M: moderate
MVIC: maximal voluntary isometric contraction
PG: Pilates group
OI: obliquus internus abdominis
OE: obliquus externus abdominis
PT: Pilates training
RMS: root mean square
SE: Standard error
T: threshold
VL: vastus lateralis
VO_2_: oxygen consumption
VO_2max_: maximum oxygen consumption
VT2: second ventilatory threshold
W: Watts
