# Correction: Pilates training improves 5-km run performance by changing metabolic cost and muscle activity in trained runners

**DOI:** 10.1371/journal.pone.0196509

**Published:** 2018-04-24

**Authors:** Paula Finatto, Edson Soares Da Silva, Alexandre B. Okamura, Bruna P. Almada, Jorge L. L. Storniolo, Henrique B. Oliveira, Leonardo A. Peyré-Tartaruga

Dr. Jorge L.L. Storniolo should be included in the author byline. He should be listed as fifth author, and his affiliation is 3: Laboratory of Locomotion Physiomechanics, Department of Pathophysiology and Transplantation, University of Milan, Italy. The contributions of this author are as follows: Conceptualization, Investigation, Methodology, Writing–Review & Editing.

The correct citation is: Finatto P, Silva ESD, Okamura AB, Almada BP, Storniolo JLL, Oliveira HB (2018) Pilates training improves 5-km run performance by changing metabolic cost and muscle activity in trained runners. PLoS ONE 13(3): e0194057. https://doi.org/10.1371/journal.pone.0194057
