# Supplementary material for: Pilates training improves 5-km run performance by changing metabolic cost and muscle activity in trained runners
Source: PLoS One. 2018 Mar 21;13(3):e0194057. doi: 10.1371/journal.pone.0194057 (PMC5862462; doi:10.1371/journal.pone.0194057)
Supplement: S1 Table — (DOCX) [file pone.0194057.s001.docx]

**Supplementary material 1**

**Table 1. 12-Week Periodization of Running Training Using the Following Intensity Scores: E, Easy; M, Moderate; T, Threshold; and I, Interval, as a Function of the Heart Rate at VT2.**

|  | **Sessions** | **1** | **2** |
| --- | --- | --- | --- |
| **Week** |  |  |  |
| **1** |  | 1 x 2500 m – E running  5 x 5 min – M running  – 1-min walking interval  1 x 1590 m – M running | 1 x 530 m – walking  3 x 1590 m – T running  – 1-min walking interval  10 min – M running |
| **2** |  | 400 m – E running  Progressive 400/800/1200 m  – 400-m interval – E running  Regressive 1200/800/400 m – 400-m interval – E running | 200 m – M running  1-min walking  1060 m – M running  1-min walking  3750 m – M running |
| **3** |  | 10 min – E running  1-min walking  30 min – M running  1-min walking  10 min – M running | 1250 m – M running  1-min walking  10 min – E running  1-min walking  4250 m – M running |
| **4** |  | 1250 m – T running  3 x 800 m – I running  (Return to the HR of E zone)  4 x 400 m – I running  (Return to the HR of M zone)  1060 m – M running | 530 m – walking  5000 m – M running  1-min walking  1000 m – I running |
| **5** |  | 1060 m – E running  2 x 1000 m – I running  (Return to HR of E zone)  1480 m – M running | 530 m – E running  4 x 800 m – I running  – 1-min rest interval  4 x 400 m – I running  – 1-min E running interval  2120 m – E running |
| **6** |  | 800 m – E running  1780 m – M running  6 x 300 m – I running  – 100-m walking interval  3030 m – E running | 1250 m – E running  20 x 100 m – I running  – 100-m walking interval  6250 m – T running |
| **7** |  | 10 min – E running  4 x 3 min – M running  – 2-min walking interval  6 x 2 min – T running  – 1-min walking interval | 5 min – E running  5 x 4 min - M running  – 1-min walking interval  25 min – M running |
| **8** |  | 1060 m – E running  25 min – M running  6 x 3 min – T running  – 1-min walking interval | 530 m – E running  4000 m – M running  6 x 1 min – T running  – 1-min walking interval |
| **9** |  | 1780 m – E running  35 min – M running  6 x 1 min – T running  – 30-s walking interval | 625 m – E running  20 min – M running  10 x 2 min – T running  – 1-min walking interval |
| **10** |  | 5 min – E running  8 x 800 m – running  (Return to HR of E zone)  25 min – M running | 930 m – walking  1200 m – T running  2 x 800 m – T running  3 x 400 m – T running  4 x 200 m – T running  – 1-min walking interval |
| **11** |  | 2 x 200 m – E running  5 x 530 m – T running  – 2-min walking interval  5 x 5 min – M running  – 1-min walking interval | 1200 m – E running  3 x 800 m – T running  – 2-min walking interval  4 x 400 m – I running  – 2-min walking interval  20 min – M running |
| **12** |  | 800 m – E running  4 x 800 m – T running  – 2-min walking interval  3 x 400 m – I running  – 2-min walking interval  15 min – M running | 800 m – E running  1200 m – T running  2-min walking  1000 m – T running  2-min walking  2 x 800 m – M running  – 1-min walking interval  3 x 400 m – T running  – 2-min walking interval |
